# Carbohydrate intake in young female cross-country skiers is lower than recommended and affects competition performance

**DOI:** 10.3389/fspor.2023.1196659

**Published:** 2023-07-17

**Authors:** Oona Kettunen, Ritva Mikkonen, Jaakko Mursu, Vesa Linnamo, Johanna K. Ihalainen

**Affiliations:** ^1^ Sports Technology Unit Vuokatti, Faculty of Sport and Health Sciences, University of Jyväskylä, Vuokatti, Finland; ^2^ Faculty of Sport and Health Sciences, University of Jyväskylä, Jyväskylä, Finland

**Keywords:** endurance sport, energy availability, body composition, FIS points, female athlete, macronutrient, nutrition, periodization

## Abstract

**Purpose:**

(1) To evaluate if energy availability (EA), macronutrient intake and body composition change over four training periods in young, highly trained, female cross-country skiers, and (2) to clarify if EA, macronutrient intake, body composition, and competition performance are associated with each other in this cohort.

**Methods:**

During a one-year observational study, 25 female skiers completed 3-day food and training logs during four training periods: preparation, specific preparation, competition, and transition periods. A body composition measurement (bioimpedance analyzer) was performed at the end of the preparation, specific preparation, and competition periods. Competition performance was determined by International Ski Federation (FIS) points gathered from youth national championships.

**Results:**

EA (36–40 kcal·kg FFM^−1^·d^−1^) and carbohydrate (CHO) intake (4.4–5.1 g·kg^−1^·d^−1^) remained similar, and at a suboptimal level, between training periods despite a decrease in exercise energy expenditure (*p* = 0.002) in the transition period. Higher EA (*r* = −0.47, *p* = 0.035) and CHO intake (*r* = −0.65, *p* = 0.002) as well as lower FM (*r* = 0.60, *p* = 0.006) and F% (*r* = 0.57, *p* = 0.011) were associated with lower (better) FIS-points. CHO intake was the best predictor of distance competition performance (*R*^2^ = 0.46, *p* = 0.004).

**Conclusions:**

Young female cross-country skiers had similar EA and CHO intake over four training periods. Both EA and CHO intake were at suboptimal levels for performance and recovery. CHO intake and body composition are important factors influencing competition performance in young female cross-country skiers.

## 1. Introduction

The annual training plan of most athletes is periodized into macrocycles (training periods), which have their own specific training and performance goals (1). The typical training year for cross-country (XC) skiers consists of a one- to three-part training period between May and November, a competition period from November to April, and a few weeks long transition period from April to May to allow for recovery before a new training year (2–5). The aim of this periodized training plan is to develop a variety of physiological, technical, and tactical capabilities that, together with competitive equipment, are needed for successful XC skiing performance (3).

Due to the variable training load and goals across training periods, nutritional needs may change. Therefore, to promote and support training goals, nutrition should also be periodized (6, 7). Periodized nutrition means that macronutrient intake is modified between training periods to support the specific training goals while maintaining adequate micronutrient intake and energy availability (EA) and avoiding negative health and performance consequences (6, 8, 9). In particular, carbohydrate (CHO) requirements may vary significantly depending on the intensity, volume and goals of the training (6). In XC skiing, most competitions and key training sessions are performed at intensities that are highly dependent on CHO based fuels (3, 10). Consequently, inadequate CHO intake may impair training intensity and competition performance, delay recovery, and increase the risk of lost training days due infection or injury (9–11).

In young athletes, the main goal of nutrition is to ensure optimal energy and nutrient availability to promote performance, recovery, training adaptations, overall health, and normal physical development (12). Physical maturation also induces changes in nutritional needs and performance capabilities, especially for young female athletes, who typically gain more body fat during adolescence between the ages of 8 and 20 years (13). As previous findings suggest that leaner body composition may confer a competitive advantage in young female XC skiers (14), more knowledge is needed, on how nutrition affects performance and body composition.

A recent study by Kettunen et al. (15) found that most young female XC skiers had suboptimal CHO intake and EA during normal training days and during an intensified 5-day training camp, despite some nutritional periodization practices. As research regarding the nutritional practices across the training year is still limited, the aim of this study was to evaluate if EA, macronutrient intake, and body composition change over four macrocycles in young female XC skiers. In addition, the study aimed to clarify how EA, macronutrient intake, body composition, and competition performance are associated with each other.

## 2. Materials and methods

### 2.1. Participants

A total of 27 female XC skiers and biathletes from a local high school sport academy (age 15–19 years) provided written informed consent to participate in this observational study. Two participants dropped out due personal reasons and thus the final number of the participants was 25. The proportion of the participants, who belonged to the youth XC or biathlon national team was 64%. Due to some missing data, the number of the participants varies between analyses, and therefore *n* values are presented in tables.

### 2.2. Design

**Figure 1:**
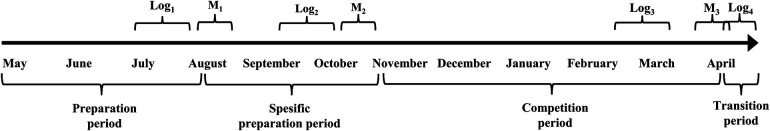
The timing of the training periods, food, and training logs (Log_1_–Log_4_), and measurements (M_1_–M_3_) during a one-year follow-up study.

Data were prospectively collected for one training year from the beginning of May to the end of April (Figure 1). The participants completed four 3-day food and training logs, one for each training period (macrocycle) of the training year as follows: preparation (Log_1_), specific preparation (Log_2_), competition (Log_3_), and transition period (Log_4_). Laboratory measurements were performed at the end of the preparation (M_1_), specific preparation (M_2_), and competition periods (M_3_). M_3_ was performed 12 ± 5 days after the Finnish Youth Championships in XC skiing, which was the main goal of the year for most of the participants. Total training volume (total hours) was recorded daily using an electronic training log (eLogger, eSportwise Oy, Finland). Competition performance was determined by International Ski Federation (FIS) points gathered from Finnish Youth National Championships. The study was approved by the ethical board of the University of Jyväskylä (20.3.2020) and conducted in accordance with the Declaration of Helsinki.

### 2.3. Anthropometric measurements (M_1_, M_2_, M_3_)

Anthropometric measurements were completed in the morning following an overnight fast. The height of each participant was measured with a wall-mounted stadiometer. Body mass (BM), fat mass (FM), fat free mass (FFM), and fat percent (F%) were measured using bioimpedance analyzer (Inbody 720, Biospace Co., Seoul, Korea). Participants were barefoot and in their underwear during the measurement, which was performed in a private room.

### 2.4. Race performance

The level of XC skier performance can be evaluated by FIS points (16, 17). FIS point calculations are based on competition performance as presented by Jones et al. (14) and lower FIS points indicate a better performance (17). As many FIS level competitions were canceled due to the COVID-19 pandemic, competition performance was evaluated by FIS points earned from Youth National Championships to which both XC skiers and biathletes took part. The best points from three distance competitions and one sprint competition were recorded separately. A total of 20 participants competed in at least one distance race and 14 participants competed in the sprint race.

### 2.5. Food and training logs

Three-day food and training logs were collected at four time points (Log_1_–Log_4_). Participants selected three subsequent days for each log from a 4-week period and were asked to select days that reflect their normal life and training as well as possible. Participants recorded the timing, type, and weight of foods and fluid consumed, quantifying their intake using kitchen scales (Idéale+, Tokmanni Oy, Mäntsälä, Finland). Verbal and written instructions were given to ensure a more accurate record keeping. Participants were instructed to take at least two photographs of the weighed portions, and whenever the scales were not available (e.g., in a restaurant) to validate what was recorded. The timing, type, and average heart rate (HR) of all exercises performed were recorded in training logs. Written and verbal instructions were given for accurate record keeping. Food logs were analyzed for energy intake (EI) and macronutrient intake using Aivodiet-software (version 2.0.2.3, Mashie, Malmö, Sweden). Although there are significant challenges in the validity of food logs, they are the best available tool for assessing dietary intake of the athletes (18).

### 2.6. Assessment of exercise energy expenditure and energy availability

The exercise energy expenditure (EEE) assessments were based on the individual relationships between HR, oxygen uptake (VO_2_) and energy expenditure (EE). An incremental exercise test was performed by walking with ski poles on a treadmill (Telineyhtymä, Kotka, Finland) during M_1_, M_2_, and M_3_. The test started at an inclination of 3.5° with a speed of 5.0 km^−1^·h^−1^. The inclination and/or speed of the treadmill was increased every third minute so that oxygen demand calculated using the equation by Balke & Ware (19) increased 6 kcal·kg FFM^−1^·d^−1^ every stage. The treadmill was not stopped between the stages. Breathing gases were measured continuously using Medikro 919 Ergospirometer (Medikro Oy, Kuopio, Finland). The average VO_2_ and respiratory exchange ratio (RER) from the last 60 s of each stage were recorded. Heart rate (HR) was monitored continuously throughout the tests using a Polar H10 HR belt (Polar Electro Oy, Kempele, Finland), and the average HR from the last 60 s of each stage was recorded. The protocol was selected as it is commonly used at local Olympic Training Center to monitor participants’ performance in a similar way as was done in the present study. Therefore, participants were familiar with the protocol.

The first five stages of the treadmill test were utilized to form an individual regression line for each participant as described by Tomten & Hostmark (20). EE during each stage was calculated as (21):

EE=VO2×(1.1×RER+3.9)

HR was strongly linearly correlated with calculated EE at increasing workloads (*r* = 0.99 at each measurement point) (21). EEE for each training session was calculated from the duration and mean HR of the training session using the regression line. The resting EE during exercise was calculated using the Cunningham equation (22) and subtracted from EEE in line with the latest definition of EA (23). Laboratory-based measures where HR is plotted against indirect calorimetry are regarded as the best methods to assess EEE in field conditions (24). Importantly, the mean intensity of most training sessions recorded were performed at an intensity between the first and fifth stage of the treadmill test.

Daily EA was calculated as (25):

$$EA=\frac{EI-EEE}{FFM}$$

where FFM is fat free body mass obtained from the bioimpedance measurement (25).

As FFM and relationship between HR and EE may change within a year, M_1_ was used to analyze Log_1_, M_2_ to analyze Log_2_, and M_3_ to analyze Log_3_ and Log_4_.

### 2.7. Statistical analyses

Statistical analyses were performed with IBM SPSS Statistics version 26.0 (IBM Corp., Armonk, NY). All data were checked for normality with a Shapiro-Wilk test and nonparametric tests were used with non-normally distributed data. The changes in variables between different parts of the training year were analyzed using a one-way analysis of variance for repeated measurements followed by the Bonferroni *post-hoc* test. Wilcoxon signed rank test was used for nonparametric variables. Results are reported as means ± SD. Correlations for normally distributed data were analyzed by using Pearson's correlation coefficient (*r*) and nonparametric data were analyzed using Spearman's correlation coefficient (*r_s_*). Stepwise linear regression analysis was used to determine which variables were the best predictors FIS points. The relative importance of predictors was calculated based on *R* Square (*R*^2^). A *p*-value <0.05 was defined statistically significant.

## 3. Results

As presented in Table 1, EI, EEE, and macronutrient intake remained similar between Log_1_, Log_2_, and Log_3_ while EI, EEE, and protein intake were lower in Log_4_ than in Log_1,_ Log_2_, and Log_3_. Nevertheless, EA remained statistically similar between the logs. Yearly training volume was 610 ± 89 h and ranged between 450 and 815 h. There were no significant differences between the daily training volume reported in the food and training logs compared to the mean training volume reported in the electronic training diaries during each training period.

**Table 1:** Food and training log data between four training periods in young female XC skiers (*n* = 23).

	Preparation period (Log_1_)	Specific preparation period (Log_2_)	Competition period (Log_3_)	Transition period (Log_4_)
Energy intake (kcal·kg FFM^−1^·d^−1^)	50.8 ± 10.6	52.5 ± 9.3	53.3 ± 8.9	46.2 ± 10.7^bb^^,^ ^cc^
Exercise energy expenditure (kcal·kg FFM^−1^·d^−1^)	14.1 ± 5.5	14.2 ± 5.3	13.3 ± 5.7	7.7 ± 6.9^aa^^,^ ^bbb^^,^ ^cc^
Energy availability (kcal·kg FFM^−1^·d^−1^)	36.7 ± 11.0	37.8 ± 10.7	40.0 ± 9.6	38.5 ± 11.2
Carbohydrate intake (g·kg^−1^·d^−1^)	5.0 ± 1.0	5.0 ± 1.1	5.1 ± 1.0	4.4 ± 1.1
Protein intake (g·kg^−1^·d^−1^)	2.0 ± 0.5	2.1 ± 0.4	2.0 ± 0.4	1.6 ± 0.4^aaa^^,^ ^bbb^^,^ ^ccc^
Fat intake (g·kg^−1^·d^−1^)	1.5 ± 0.5	1.5 ± 0.4	1.5 ± 0.4	1.3 ± 0.5
Training (food diaries) (h·d^−1^)	2.1 ± 0.7	2.1 ± 0.7	1.9 ± 0.7	1.1 ± 0.7^aaa^^,^ ^bb^^,^ ^cc^
Training (eLogger) (h·d^−1^)	1.9 ± 0.3	1.8 ± 0.3	1.5 ± 0.5^aa^^,^ ^bb^	0.5 ± 0.4^aaa^^,^ ^bbb^^,^ ^ccc^

^aa^ Significantly different from preparation period (Log_1_) *p* < 0.01.

^aaa^ *p* < 0.001.

^b^ Significantly different from specific preparation period (Log_2_) *p *< 0.05.

^bb^ *p* < 0.01.

^bbb^ *p* < 0.001.

^c^ Significantly different from competition period (Log_3_) *p* < 0.05.

^cc^ *p *< 0.01.

^ccc^ *p *< 0.001.

As presented in Table 2, BM and body mass index (BMI) increased from M_1_ to M_2_ and further from M_2_ to M_3_. In addition, FM and F% increased from M_2_ to M_3_.

**Table 2:** Anthropometric variables in the end of three macrocycles in young female XC skiers.

	*n*	M_1_ (August)	M_2_ (November)	M_3_ (April)
Body mass (kg)	24	61.8 ± 6.8	63.1 ± 6.8^aaa^	64.7 ± 7.3^aaa^^,^ ^bb^
Height	24	168.2 ± 5.2	168.5 ± 5.3	168.6 ± 5.2
BMI (kg·m^−2^)	24	21.8 ± 2.2	22.2 ± 2.2^aaa^	22.7 ± 2.7^aaa^^,^ ^bb^
Fat free mass (kg)	23	51.0 ± 5.2	51.9 ± 5.0	51.5 ± 4.9
Fat mass (kg)	23	10.6 ± 3.6	10.9 ± 4.0	13.0 ± 4.3^aaa^^,^ ^bbb^
Fat percent (%)	23	17.0 ± 4.8	17.1 ± 5.2	20.0 ± 5.0^aaa^^,^ ^bbb^

^a^ Significantly different from M_1_ *p* < 0.05.

^aa^ *p* < 0.01.

^aaa^ *p* < 0.001.

^b^ Significantly different from M_2_ *p* < 0.05.

^bb^ *p* < 0.01.

^bbb^ *p* < 0.001.

Mean FIS distance points for participants from the Youth National Championships were 171.6 ± 52.0 (*n* = 20, range 101.0–274.6) and mean FIS sprint points were: 186.2 ± 32.0 (*n* = 14, range 127.2–239.6).

As presented in Table 3, better success in XC distance races, indicated by lower FIS distance points, was associated with higher EA and macronutrient intake as well as with lower FM and F%. FIS sprint points were negatively associated with training volume. In addition, athletes with higher FM and F% tended to eat less CHO, protein, and fat in relation to their BM (Table 3). When stepwise linear regression was performed using the variables presented in Table 3, CHO intake explained 46% of the variance in FIS distance points (*R*^2^ = 0.46, *p* = 0.004). Training volume was the best predictor for FIS sprint points (*R*^2 ^= 0.34, *p *= 0.036).

**Table 3:** Correlation coefficients (*r_s_* for BM, *r* for others) between FIS points, EA, and macronutrient intake (the mean of Log_1_, Log_2_ and Log_3_), anthropometrics in the end of the competition season, and yearly training volume.

	FIS_d_	FIS_s_	EA	CHO	Protein	Fat	BM	FFM	FM	F%	Training
FIS_d_	1										
FIS_s_	0.36	1									
EA	−0.47*	−0.23	1								
CHO	−0.65**	0.01	0.69***	1							
Protein	−0.51*	0.22	0.53**	0.75***	1						
Fat	−0.53*	0.31	0.82***	0.69***	0.65**	1					
BM	0.42	−0.02	−0.23	−0.28	−0.08	−0.27	1				
FFM	0.28	−0.13	−0.38	−0.16	0.11	−0.17	0.83***	1			
FM	0.60**	0.09	−0.37	−0.60**	−0.60**	−0.49*	0.77***	0.28	1		
F%	0.57*	0.17	−0.20	−0.55**	−0.64**	−0.43*	0.51*	−0.06	0.93***	1	
Training	−0.27	−0.58*	−0.31	−0.29	−0.31	−0.09	0.07	−0.02	0.13	0.11	1

FIS_d_, FIS distance points; FIS_s_, FIS sprint points; EA, energy availability (kcal·kg FFM^−1^·d^−1^); CHO, carbohydrate intake (g·kg^−1^·d^−1^); BM, body mass; FFM, fat free body mass (kg); FM, fat mass (kg); F%, fat percent.

* *p* < 0.05.

** *p* < 0.01.

*** *p* < 0.001.

## 4. Discussion

The present study assessed the changes in nutritional intake and body composition during a training year in young female XC skiers. The results showed that athletes had similar EEE, EI, EA, and macronutrient intake during preparation, specific preparation, and competition periods. However, athletes decreased EI during the transition period, where they also experienced lower EEE, thus maintaining EA. Unfortunately, in most athletes, CHO and EA were lower than recommended for performance and training adaptations (9). The second aim of the study was to assess the relationships between nutrition, body composition, and competition performance. Interestingly, better performance in XC distance competitions was associated with higher EA and macronutrient intake as well as with lower FM and F%. CHO intake was the best predictor for FIS distance points.

The EEE and training volume between preparation (Log_1_), specific preparation (Log_2_), and competition periods (Log_3_) remained similar but decreased significantly in the transition period (Log_4_). Also nutritional requirements of the training remained quite similar during the first three training periods, where athletes had similar EI, EA, and macronutrient intake. Nevertheless, during the transition period, athletes adapted to smaller energy needs by consuming less energy. Therefore, athletes seemed to periodize their EI between transition period and other training periods. Indeed, EA remained stable despite the variation in energy needs, which is in line with periodized nutrition recommendations (6). This finding is also in line with the findings of Ihalainen et al. who found that young female runners had similar EA in different parts of their training year (26). Unfortunately, the data of the present study does not reveal whether the lower EI during transition period was intentional or spontaneous.

The mean EA of athletes during the present one-year follow-up varied between 36 and 40 kcal·kg FFM^−1^·d^−1^, which is considered suboptimal for performance and training adaptations but adequate to maintain normal physiological functions (25). The EA detected in the present study is similar to that reported in young female XC skiers at home and training camp conditions (15) and in young female distance runners during different parts of the training year (26). Notably, the individual variation was high, and six athletes had low EA (<30 kcal·kg FFM^−1^·d^−^1) in Log_1_, while four had low EA in Log_2_, two in Log_3_, and five in Log_4_. In the long term, these individuals may be at risk to develop deleterious health and performance consequences related to low EA such as hormonal disturbances, decreased bone health, and increased risk of injury (8, 27).

When comparing the results from the first three food logs to the nutrition recommendations for endurance athletes training 1–3 h·d^−1^, protein intake (2.0–2.1 g·kg^−1^·d^−1^) was in the upper limits (1.2–2.0 g·kg^−1^·d^−1^), fat intake (1.5 g·kg^−1^·d^−1^ i.e., −31% of total EI) was in the recommended range (20%–35% from total EI), and CHO intake (5.0–5.1 g·kg^−1^·d^−1^) was less than recommended (6–10 g·kg^−1^·d^−1^) (9). These findings are similar to those previously reported in Swedish national team XC skiers (28) and in Finnish Youth National Team XC skiers (15). Thus, it seems that consuming adequate CHO is a notable challenge for female XC skiers of different ages and nationalities.

BM and F% increased significantly over the training year and especially during the competition period, which is against the body composition periodization principles that are used to optimize performance in elite athletes (29). Importantly, an increase in BM, FM, and F% is also a normal physiological phenomenon during adolescence (13), which may explain, at least partly, why BM, FM, and F% increased as the study progressed. BM increased evenly from M_1_ to M_3_, while the increase from M_1_ to M_2_ was mostly explained by the increase in FFM while the increase from M_2_ to M_3_ is explained by an increase in FM, which led to a simultaneous increase in F%.

Better competition performance in distance events at the Youth National Championships was associated with higher EA and macronutrient intake as well as with lower FM and F%. CHO intake was the best predictor for FIS distance points. The present results indicate that adequate CHO intake across the training year may have an important role in optimizing competition performance. This finding is logical, as inadequate CHO intake may impair training intensity and competition performance, delay recovery and increase the risk of lost training days due infection or injury (9–11).

In addition to the findings that nutritional intake and body composition were associated with performance, we found that anthropometric and nutritional variables correlated with each other. Interestingly, higher FM and F% were associated with lower intake of all macronutrients. Because of the observational study setting, reliable causal relationships cannot be determined. One potential explanation may be that athletes with higher body fat restricted their eating to lose BM without desired results. Another explanation may be that higher body fat is an adaptation to chronic low EA as long-term low EA leads to energy conserving metabolic changes that, over the long term may become detrimental to training adaptations and sport performance (8, 27). It is also possible that athletes who have adopted proper eating practices that support their training, have succeeded in developing their performance from year to year whereby their body composition has adapted to the demands of their sport due to successful training and/or genetics. It is worth noting, that the macronutrient intake in the present study and current nutritional recommendations (9) are expressed in relation to BM. Athletes with higher FM and F% have higher amount of metabolically inactive tissue and therefore, it is possible that they do not need as much fuel in relation to their BM as their leaner counterparts.

FIS sprint points were predicted by yearly training volume but were not associated with any other variables. Similar results were reported by Jones et al. (14) who did not find any predictors for FIS sprint points. In contrast, Carlsson et al. (30) found that lean body mass predicted XC sprint performance in elite female skiers. It is difficult to assess why training volume predicted sprint but not distance performance in present study. Nevertheless, it is important to note that only 14 participants took part in the sprint race at Youth Nationals, which increases the risk of error in FIS sprint point analyses, thus these results should be interpreted with caution.

The present study has some limitations. One of the major limitations is that the methods used to assess dietary intake and EA in field conditions are prone to errors (24, 31). To minimize these errors, we selected methods that are considered the most valid to assess EEE and dietary intake in field conditions (24). Food and training logs were completed during different parts of the training year, which gave more reliable information regarding overall practices. Notably, training logs reflected the training volume expected from each training period suggesting that training logs reflected actual training completed. Finally, FIS points were recorded only from single races instead of a yearly score. Nevertheless, we believe that this gave the best possible description of the competition performance as many races were cancelled due to the COVID-19 pandemic and the Youth National Championships were among the most important competitions for our participants. Importantly, it should be recognized that non-physiological factors such as ski selection, waxing, and environmental factors may have affected competition performance and FIS points.

### 4.1. Conclusions

Young female XC skiers maintained similar, but suboptimal, EA and carbohydrate intake between four training periods. EEE remained similar between preparation, specific preparation, and competition periods suggesting similar dietary requirements during these macrocycles. Nevertheless, athletes experienced lower EEE during the transition period, but maintained EA by decreasing EI. CHO intake across the training year seems to be an important predictor for XC distance competition performance in young female XC skiers. Furthermore, lower BM, FM, and F% may be beneficial for distance competition performance. Based on our results, however, restricting dietary intake may not be an optimal way to modify body composition in young female athletes. Therefore, based on the findings of present study, young female XC skiers should aim to maintain adequate EA and CHO intake throughout the training year to promote long-term development in competition performance.

## Data Availability

The raw data supporting the conclusions of this article will be made available by the authors, without undue reservation.
